# Alzheimer’s disease genetics and real‐world patient data fuel target and drug discovery in European and African American Ancestries using AI/ML approaches

**DOI:** 10.1002/alz.087887

**Published:** 2025-01-03

**Authors:** Feixiong Cheng, Yuan Hou, Pengyue Zhang, Fan Fan, Jonathan L. Haines, Andrew A. Pieper, Cindy McReynolds, Bruce D. Hammock, James B Leverenz, Jeffrey L. Cummings

**Affiliations:** ^1^ Cleveland Clinic, Cleveland, OH USA; ^2^ Indiana University, Bloomington, IN USA; ^3^ Augusta University, Augusta, GA USA; ^4^ Cleveland Institute for Computational Biology, Case Western Reserve University, Cleveland, OH USA; ^5^ Department of Genetics and Genome Sciences, Case Western Reserve University School of Medicine, Cleveland, OH USA; ^6^ Case Western Reserve University School of Medicine, Department of Population & Quantitative Health Sciences, Cleveland Institute for Computational Biology, Cleveland, OH USA; ^7^ Department of Population and Quantitative Health Sciences, Institute for Computational Biology, Case Western Reserve University, Cleveland, OH USA; ^8^ Case Western Reserve University, Cleveland, OH USA; ^9^ Eicosis Inc., Davis, CA USA; ^10^ University of California, Davis, Davis, CA USA; ^11^ Cleveland Clinic Lou Ruvo Center for Brain Health, Cleveland, OH USA; ^12^ Chambers‐Grundy Center for Transformative Neuroscience, Department of Brain Health, School of Integrated Health Sciences, University of Nevada Las Vegas, Las Vegas, NV USA

## Abstract

**Background:**

Although high‐throughput DNA/RNA sequencing technologies have generated massive genetic and genomic data in human disease, translation of these findings into new patient treatment has not materialized by lack of effective approaches, such as Artificial Intelligence (AL) and Machine Learning (ML) tools.

**Method:**

To address this problem, we have used AI/ML approaches, Mendelian randomization (MR), and large patient’s genetic and functional genomic data to evaluate druggable targets using Alzheimer’s disease (AD) as a prototypical example. We utilized the genomic instruments from 9 expression quantitative trait loci (eQTL) and 3 protein quantitative trait loci (pQTL) datasets across five human brain regions from three biobanks. We tested the outcome of Mendelian randomization across genome‐wide association studies (GWAS) datasets of European‐American (EA) and African‐American (AA) ancestries, with 275,540 AD cases and 1.55 million controls. We searched repurposable drugs using AI‐assistant drugome‐wide association studies from ∼80 million electronic health records.

**Result:**

We identified 25 drug targets in EAs and 6 new drug targets in AAs. Among 6 AA‐specific targets, TRPV3 is a potent drug target and replicated in AA‐specific eQTL data from the Metabrain cohort. We pinpointed that an anti‐inflammatory AD target of epoxide hydrolase 2 (EPHX2): (1) a pQTL lead SNP rs2741342 (P_GWAS_ = 5.72 × 10^‐13^; P_pQTL_ = 1.19 × 10^‐16^) located in an enhancer of *EPHX2*; and (2) a protein‐coding variant of rs751141 (p.Arg287Gln) was associated with reduced EPHX2 protein expression (P_pQTL_ = 5.50 × 10^‐16^) from the AD knowledge portal. We demonstrated that TPPU (a nanomolar‐EPHX2 inhibitor) blocked deterioration in hippocampal‐dependent cognitive ability in a TgF344‐AD rat model and EC5026 (a first‐in‐class, picomolar EPHX2 inhibitor) improves cognition in a 5xFAD mouse model. We identified 23 candidate drugs associated with reduced risk of AD in mild cognitive impairment patients. We found that usage of either apixaban (hazard ratio [HR] = 0.74, 95% confidence interval [CI] 0.69–0.80) and amlodipine (HR = 0.91, 95%CI 0.88–0.94) were significantly associated with reduced progression to AD.

**Conclusion:**

Combining genetics and real‐world patient data identifies ancestry‐specific therapeutic targets and medicines for AD. Further functional and clinical validation of candidate targets and drugs in ethnically diverse population are warranted.

